# Commentary on: Inspiratory Muscle Training, with or without Pulmonary Rehabilitation, for COPD: A Critical Appraisal of a Cochrane Review

**DOI:** 10.56792/WUHG8221

**Published:** 2025-04-23

**Authors:** D Holland, E Porter, C Tait, J E Hill, C Harris

**Affiliations:** Blackpool Teaching Hospitals NHS Foundation Trust; University of Lancashire

## Introduction

Approximately 300 million people worldwide have COPD (1) with a global prevalence of between approximately 10-12.2% (2–4). COPD is a highly prevalent respiratory disease in the United Kingdom, ranking as the second most common lung disease, with approximately 1.2 million people diagnosed (5).

In COPD, oxidative stress and sarcomere injury lead to proteolysis and subsequent atrophy of the diaphragm, the main inspiratory muscle (6). The reduction in capacity of the respiratory muscles lead to a reduction in the body’s capacity to generate inspiratory pressures, thus reducing lung capacity and is thought to contribute to dyspnoea in adults with advanced COPD (7).

Respiratory muscle weakness is a modifiable weakness and can be targeted with interventions including variations in frequency and duration of Inspiratory Muscle Training (IMT) (8). Inspiratory muscle training uses resistance to challenge inhalation, stimulating respiratory muscles and potentially enhancing contractile force through hypertrophy (9). A previous Cochrane review examined the effectiveness of IMT, both with and without pulmonary rehabilitation, in increasing inspiratory muscle strength in individuals with COPD (10).

## Aim of Commentary

This commentary aims to critically appraise the methods used within the review by Ammous et al., (2023) and expand upon the findings in the context of clinical practice.

## Critical Appraisal and Methods of Ammous et al., (2023)

Using the AMSTAR 2 tool this review achieved 16 out of 16 criteria (see Table 1 for full results and corresponding methods). Therefore, the methods used in the review were appropriate in context to answering the question of interest.

## Results of Ammous et al., (2023)

When comparing IMT to control or sham treatment in people with COPD there was a statistically significant improvement in:

- Dyspnoea scores
  ○ Borg scale and BDI-TDI (very low-certainty)
  ○ Modified Medical Research Council (mMRC) Scale (low-certainty: Clinically significant improvement)
- Functional exercise capacity (moderate-certainty: Clinically significant improvement)
- Inspiratory muscle strength (low-certainty)
- Health-related quality of life
  ○ COPD Assessment Test (CAT) scale (moderate-certainty: Clinically significant improvement)

However, there was no evidence of difference (very low certainty evidence) for health-related quality of life when using the St. George’s Respiratory Questionnaire (SGRQ) total score.

There was no evidence of a difference between pulmonary rehabilitation combined with IMT compared to pulmonary rehabilitation alone in people with COPD for:

- Dyspnoea
  ○ Borg scale (moderate-certainty)
  ○ mMRC scale (very low-certainty)
- Functional exercise capacity (very low-certainty)
- Health-related quality of life
  ○ SGRQ total score (low-certainty)
  ○ CAT scale (very low-certainty)

There was moderate certainty evidence that pulmonary rehabilitation combined with IMT compared to pulmonary rehabilitation alone produced a small statistically significant increase in inspiratory muscle strength. The subgroup analyses showed no evidence of a difference regarding the duration of intervention variables or the severity of respiratory muscle weakness when comparing pulmonary rehabilitation plus IMT to pulmonary rehabilitation alone, or IMT to control or sham treatment.

## Commentary

The findings from this review suggest that there is low-certainty evidence indicating that IMT may lead to a slight increase in inspiratory muscle strength in patients with COPD compared to control or sham treatment. However, there seems to be low to moderate certainty evidence suggesting that IMT leads to a clinically significant improvement in dyspnoea (mMRC), functional exercise capacity, and health-related quality of life (CAT scale) compared to control or sham treatment. Previous systematic reviews in this area have found similar findings regarding the effects of IMT on inspiratory muscle strength, dyspnoea, functional exercise capacity, and health-related quality of life (11). Furthermore, these findings are in line with recommendations within the gold standard for COPD (2). It is recommended when designing a pulmonary rehabilitation programme that IMT should be considered as a possible approach used within the programme (2). Furthermore, IMT should be considered for patients with hyperinflation as hyperinflation correlates closely with diffusion capacity, small airways obstruction and higher ventilatory response to exercise (2). In terms of delivering IMT, the systematic review found no evidence of significant moderating factors regarding the duration of the intervention or whether individuals with greater inspiratory muscle weakness would particularly benefit from this approach. Guidance suggests that pulmonary rehabilitation should be offered for individuals with a minimum of a Medical Research Council [MRC] Grade 3 and above (12) and that programs should last from six to eight weeks (2, 12).

As highlighted in this review, when combined with pulmonary rehabilitation IMT does not appear to provide additional benefit outside a slight improvement in inspiratory muscle strength. These findings support historical recommendations from the American Thoracic Society/European Respiratory Society that IMT may not provide much benefit when delivered as an adjunct to standard pulmonary rehabilitation (13). However, for those individuals who may not be able to attend exercise based pulmonary rehabilitation IMT may be considered as a possible alternative.

Most estimates in this review were downgraded due to methodological issues in the included RCTs. Future trials should clearly report randomisation sequencing and allocation concealment, as many studies were rated at a high risk of bias due to unclear methods. Adopting CONSORT checklist standards can help ensure transparent reporting of future studies (14). It is worth noting that most studies in this review excluded participants who experienced exacerbations prior to the trial. This exclusion significantly limits the external validity of the findings for this population. Consequently, further investigation of IMT is needed specifically within this group. Given the uncertainty in the estimates, future RCTs should assess both dyspnoea and inspiratory muscle strength outcomes. Additionally, substantial heterogeneity in muscle strength gains when comparing IMT to control suggests the need to evaluate key moderating factors in future trials. Neither intervention duration nor baseline muscle weakness explained this heterogeneity. Future systematic reviews may also consider using multifactorial meta-regression to identify the sources of unexplained variation in effect.
